# Using the Consolidated Framework for Implementation Research to Identify Barriers and Facilitators for the Implementation of an Internet-Based Patient-Provider Communication Service in Five Settings: A Qualitative Study

**DOI:** 10.2196/jmir.5091

**Published:** 2015-11-18

**Authors:** Cecilie Varsi, Mirjam Ekstedt, Deede Gammon, Cornelia M Ruland

**Affiliations:** ^1^ Center for Shared Decision Making and Collaborative Care Research Oslo University Hospital Oslo Norway; ^2^ Faculty of Medicine University of Oslo Oslo Norway; ^3^ Royal Institute of Technology School of Technology and Health Stockholm Sweden; ^4^ Norwegian Centre for Integrated Care and Telemedicine University Hospital in North Norway Tromsø Norway

**Keywords:** implementation, Internet, electronic mail, secure Web communication, eHealth, qualitative research, Consolidated Framework for Implementation Research, CFIR

## Abstract

**Background:**

Although there is growing evidence of the positive effects of Internet-based patient-provider communication (IPPC) services for both patients and health care providers, their implementation into clinical practice continues to be a challenge.

**Objective:**

The 3 aims of this study were to (1) identify and compare barriers and facilitators influencing the implementation of an IPPC service in 5 hospital units using the Consolidated Framework for Implementation Research (CFIR), (2) assess the ability of the different constructs of CFIR to distinguish between high and low implementation success, and (3) compare our findings with those from other studies that used the CFIR to discriminate between high and low implementation success.

**Methods:**

This study was based on individual interviews with 10 nurses, 6 physicians, and 1 nutritionist who had used the IPPC to answer messages from patients.

**Results:**

Of the 36 CFIR constructs, 28 were addressed in the interviews, of which 12 distinguished between high and low implementation units. Most of the distinguishing constructs were related to the inner setting domain of CFIR, indicating that institutional factors were particularly important for successful implementation. Health care providers’ beliefs in the intervention as useful for themselves and their patients as well as the implementation process itself were also important. A comparison of constructs across ours and 2 other studies that also used the CFIR to discriminate between high and low implementation success showed that 24 CFIR constructs distinguished between high and low implementation units in at least 1 study; 11 constructs distinguished in 2 studies. However, only 2 constructs (patient need and resources and available resources) distinguished consistently between high and low implementation units in all 3 studies.

**Conclusions:**

The CFIR is a helpful framework for illuminating barriers and facilitators influencing IPPC implementation. However, CFIR’s strength of being broad and comprehensive also limits its usefulness as an implementation framework because it does not discriminate between the relative importance of its many constructs for implementation success. This is the first study to identify which CFIR constructs are the most promising to distinguish between high and low implementation success across settings and interventions. Findings from this study can contribute to the refinement of CFIR toward a more succinct and parsimonious framework for planning and evaluation of the implementation of clinical interventions.

**ClinicalTrial:**

Clinicaltrials.gov NCT00971139; http://clinicaltrial.gov/ct2/show/NCT00971139 (Archived by WebCite at http://www.webcitation.org/6cWeqN1uY)

## Introduction

Internet-based patient-provider communication (IPPC) services provide patients and their health care providers with the opportunity for secure email contact over the Internet and can be a valuable supplement to traditional health services [1,2]. An increasing number of studies indicate that IPPC services can help patients manage their illness better, improve health outcomes [3-5], address unmet communication needs in health care [6-8], and improve quality of care [8,9]. Health care providers who have used IPPC services to communicate with their patients report a high level of positive attitude [7,9,10]. They find IPPC services convenient and useful for selected patients [1], and perceive them as a safe and efficient way of communicating with patients [1,10]. No studies were found that reported any harmful effects for either patients or health care providers [1,11], and utilization of IPPCs is increasingly becoming part of health care policies [12].

Despite the growing evidence that the use of IPPC services has positive outcomes for patients and health care providers [1], studies also report challenges when making use of IPPC services in clinical practice. This new form of patient-provider communication has raised health care provider concerns regarding integration of the tools into daily working routines [13] and increased workload [10,11,14]. Health care providers have been concerned that patients would pose questions not suitable for this form of communication, such as urgent or sensitive themes [15]. Concerns about technology, confidentiality, security, and liability have also been raised [7,15].

Studies have illuminated what is needed for successful implementation of evidence-based practice [16], health care improvement [17], and new technology [18] into clinical practice. Until now, studies within eHealth have focused on patient and provider outcomes and effectiveness, but have had less emphasis on understanding *why* interventions succeed or fail [19]. In this study, *eHealth* is defined as “the transfer of health resources and health care by electronic means” [20]. A recent review argues that attention should be given now to the development and evaluation of strategies to implement effective eHealth initiatives, rather than to further strengthen the evidence of effectiveness that is already available [21]. Furthermore, it is recommended to include several sites in implementation studies because an apparently similar intervention may be implemented and accepted in different ways in different settings [22] and different professions may have varying perceptions about implementation success [23]. Yet few studies report on the factors relevant for successful implementation of tools such as IPPC services in clinical practice [10], across settings, which is important to increase transferability of results.

Theoretical frameworks in implementation studies are underused [24] and evaluations of eHealth implementation require good theoretical frameworks. Use of theory in implementation studies can help identify factors that predict the likelihood of implementation success and help develop better strategies to achieve more successful implementation, thus strengthening the understanding and explanation of how and why implementation succeeds or fails (eg, what works, for whom, under what circumstances, and why) [25]. Theories, frameworks, and models can help identify appropriate outcomes, measures, and variables of interest for implementation studies. Theory can also help organize studies when collecting, analyzing, interpreting, explaining, and presenting data [26]. In preparation for this study, which identified barriers and facilitators influencing the implementation of an IPPC service in 5 hospital units, the appropriateness of several theories and frameworks was assessed. A number of implementation frameworks and theories exist and were considered, such as the Reach Effectiveness Adoption Implementation Maintenance (RE-AIM) framework [27], the Promoting Action on Research Implementation in Health Services (PARiHS) framework [28], Technology Acceptance Model [29], and Normalization Process Theory [30]. The Consolidated Framework for Implementation Research (CFIR) [31] was chosen based on its comprehensiveness and ability to manage both breadth and depth of data for capturing the complexity of IPPC implementation. The meta-theoretical basis of CFIR includes a broad number of aspects related to implementation and is thus considered a helpful framework for illuminating barriers and facilitators influencing IPPC implementation. CFIR is derived from 19 theories about dissemination, innovation, organizational change, implementation, knowledge translation, and research uptake [31]. CFIR synthesizes the spectrum of terminologies, definitions, and constructs into a consolidated framework. It is described as a “determinant framework” meaning that it specifies determinants that can act as barriers and facilitators to influence implementation outcomes [25]. CFIR is described as well-suited for implementation research on health service delivery [31,32]. It addresses the need to assess and maximize the effectiveness of implementation within a specific context and to promote dissemination to other contexts. CFIR comprises 39 constructs sorted under 5 domains [31]: (1) intervention characteristics, (2) outer setting, (3) inner setting, (4) characteristics of individuals, and (5) process.

An increasing number of implementation studies have used CFIR, some as an evaluation framework [24,32-36], some for detecting factors influencing implementation [32,37,38], and some for classifying these influencing factors as facilitators or barriers [34,35,39]. To date, only a few studies have had the evaluation of CFIR as a specific aim [40-42]. So far, we found only one other study that used the CFIR developers’ method to identify and compare distinguishing constructs between high versus low implementation settings [43]. Therefore, there was a need for more studies to assess and further develop CFIR’s applicability in explaining what factors influence implementation success. Furthermore, in CFIR, all constructs have equal weight and the framework does not distinguish between the relative importance of its different constructs. It would, therefore, add strength to the framework to increase the predictive ability of CFIR’s different constructs to discriminate between implementation success, so that the framework could become more succinct, parsimonious, and thus more easily applicable.

The aim of this study was threefold: (1) to identify and compare barriers and facilitators influencing the implementation of IPPC in 5 hospital units using CFIR as the conceptual framework, (2) to assess the ability of the different constructs of CFIR to distinguish between high and low implementation success, and 3) to compare our findings with those from other studies that have used CFIR to discriminate between high and low implementation success.

## Methods

### Intervention

**Figure 1:**
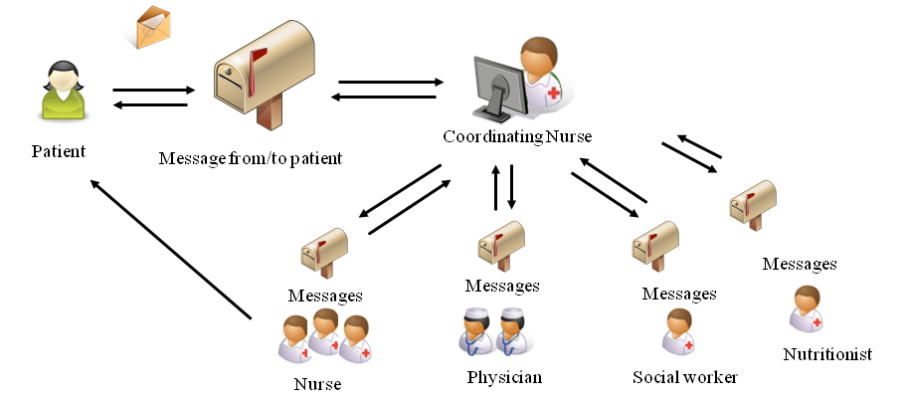
Internet-based patient-provider communication (IPPC) message flow between patients and health care providers.

The IPPC service in this study was an Internet-based communication service in which patients could send messages to and receive answers from hospital nurses, physicians, nutritionists, and social workers. The IPPC system had a high security level, requiring both patients and health care providers to log in the system by means of strong authentication keys. The message from the patient was received in the mailbox of the coordinating nurse, who had expertise on the respective diagnoses and treatments and had access to the patients’ medical record at the hospital. The nurse could address the question directly or forward the message to the mailbox of another provider who was in a better position to answer the question. This health care provider could either answer the patient directly or give comments back to the coordinating nurse, who formulated an answer to the patient (see Figure 1). The development and testing of an IPPC service is described elsewhere [44].

### Procedures

This paper reports on the identification of barriers and facilitators influencing the implementation of IPPC in 5 units treating patients with cancer or diverse medical diagnoses at a university hospital in Norway. Analysis of messages and interviews with patient users and nonusers has been reported elsewhere [6,45,46].

After initial information meetings between the unit management at each unit and the head of the research center, an agreement for implementing the IPPC service as part of a research study was signed. IPPC was not a mandatory intervention within the hospital, but was voluntarily implemented as part of routine care at these 5 units. The first step of the implementation was that the management of the units designated in total 16 nurses, 8 physicians, 4 social workers, and 1 nutritionist to the implementation and use of IPPC. Next, all contributing health care providers received thorough information about the IPPC service and one-on-one training on how to operate the system. In addition, the nurses received training on how to refer eligible patients to the research assistants for potential study inclusion, who subsequently introduced the patients to IPPC, asked for informed consent, and filled out the demographic and baseline questionnaires. The patients received a brief introduction with information about how to log in and use the IPPC service. They were informed that they could send messages with questions and concerns related to their illness and would receive advice and support from hospital health care providers in between and after their hospital admissions. The patients were informed that they could use the IPPC service as much as they wanted over the study period, which lasted for 6 or 8 months dependent on unit affiliation [45].

At each unit, the health care providers agreed among themselves who should hold the different roles in answering the patients’ messages (Figure 1) and set up routines to fill in for one another when absent. Routines were pilot-tested to streamline recruitment procedures and solve initial technical problems with the IPPC service, and this was followed up with health care provider interviews 1 month after start-up. Only small insignificant obstacles that already had been solved were found. Throughout the study, participating health care providers and unit managers received monthly emails with the number of patients recruited so far and the number of received and answered messages in the IPPC system. The research team was also available if technical IPPC system problems occurred.

During the study period of 19 to 23 months (dependent on unit), 38 of 171 patients included in the study (22%) sent 133 messages (range 1-13). The health care providers wrote 133 responses to the patients (range 0-27). Four units identified and offered more than approximately 60% of their eligible patients to the IPPC service. The fifth unit, however, did not offer more than approximately 15% to the IPPC service. Based on the unit differences in the proportion of available patients who were offered information about IPPC, we labeled the 4 units as *high implementation units* and the fifth unit as a *low implementation unit*.

### Study Design and Participants

To identify barriers and facilitators influencing the implementation of IPPC, we conducted a qualitative study [47] based on individual interviews or written feedback from nurses, physicians, and a nutritionist at the end of the IPPC implementation period. The study was planned and performed in compliance with the principles outlined in the Declaration of Helsinki [48] and was approved by the Regional Committee for Medical and Health Research Ethics in Norway and the Privacy Protection Committee at Oslo University Hospital. Written informed consent was obtained from all participants.

Ten nurses, 6 physicians, and 1 nutritionist from the 5 units answered messages from the patients in the IPPC and were, therefore, included in the interview study. One of the respondents preferred to receive and answer interview questions in writing, whereas the others participated in individual interviews. The nurses were a mean age of 40 (median 40, range 26-55) years with a mean of 16 (median 13, range 3-32) years nursing practice since graduating from nursing school and a mean of 11 (median 7, range 0.5-32) years of experience with the current diagnostic patient group. Half had a clinical specialization in nursing. The physicians and nutritionist were a mean age of 50 (median 54, range 41-58) years with a mean of 23 (median 22, range 15-31) years since graduating from medical/nutritionist school and a mean of 14 (median 16, range 2-23) years of experience with the patient diagnostic group. Five respondents among the physicians and the nutritionist had a PhD degree, 4 had clinical specialization, and 3 had both. Most respondents were women (13/17, 76%; Clinical Trial NCT00971139).

### Interview Procedure

A semistructured interview guide was developed containing questions based on the 5 domains of CFIR [31]. The 39 constructs of CFIR supported the research team in defining topics for the interviews and ensured that all major domains in the framework that influence implementation were addressed. Operationalization of CFIR domains for the current study were:

1. Intervention characteristics: the IPPC service
2. Inner setting: 5 units treating patients with cancer or diagnoses within internal medicine
3. Outer setting: the patients who were offered IPPC
4. Characteristics of individuals involved: the nurses, physicians, and nutritionist who operated the IPPC service
5. Process: the process when IPPC was implemented

The interviews were conducted by the first author either at the interviewees’ office or at a meeting room at the hospital based on the interviewee’s preference. The interviews lasted between 10 and 75 minutes; they were recorded with a digital voice recorder and transcribed verbatim, except for one of the interviews in which notes were taken by the interviewer during the interview because the respondent did not allow use of a voice recorder.

### Analysis

The transcripts were analyzed using techniques of qualitative content analysis, inspired by a deductive directed approach, deemed applicable because we wanted to analyze our data in light of an existing framework [49]. The analysis was performed by the first (CV) and the second authors (ME) in a stepwise interactive process. The first step in the analysis, after reading all transcripts, notes, and written responses to obtain an understanding of the whole, was to develop initial coding nodes and subnodes based on the domains and constructs of the CFIR framework [31]. In the second step, units of analysis, such as sentences or longer semantic units, were deductively coded into the nodes and subnodes. Third, the coded text was then subjected to a rating process based on the recommended method described by Damschroder and Lowery [50], the authors of CFIR. In the rating process, a deliberated consensus process was used to assign a rating to each construct obtained from each hospital unit. The ratings reflected the valence (positive or negative influence) and the magnitude or strength of each construct that emerged in each hospital unit based on the coded text. When all constructs obtained from all hospital units were rated, we compared ratings for each construct across hospital units [50]. Constructs were coded either as missing too much data to discern a pattern (missing), not distinguishing between high and low implementation units (0), or weakly (+1/-1) or strongly (+2/-2) distinguishing low from high implementation units. Table 1 provides definitions of the criteria used to guide assignments of the ratings. For more details, see the method paper of the CFIR developers [50].

**Table 1:** Criteria used to assign ratings to constructs.

Rating	Criteria
–2	The construct is a negative influence in the organization, an impeding influence in work processes, and/or an impeding influence in implementation efforts. The majority of respondents describe explicit examples of how the key or all aspects (or the absence) of a construct manifests itself in a negative way.
–1	The construct is a negative influence in the organization, an impeding influence in work processes, and/or an impeding influence in implementation efforts. Respondents make general statements about the construct manifesting in a negative way but without concrete examples: (1) the construct is mentioned only in passing or at a high level without examples or evidence of actual, concrete descriptions of how that construct manifests; (2) there is a mixed effect of different aspects of the construct but with a general overall negative effect; (3) there is sufficient information to make an indirect inference about the generally negative influence; and/or (4) judged as weakly negative by the absence of the construct.
0	A construct has neutral influence if: (1) it appears to have neutral effect (purely descriptive) or is only mentioned generically without valence; (2) there is no evidence of positive or negative influence; (3) credible or reliable respondents contradict each other; and/or (4) there are positive and negative influences that balance each other out, the construct has some positive influence whereas other influences are negative and, overall, the effect is neutral.
+1	The construct is a positive influence in the organization, a facilitating influence in work processes, and/or a facilitating influence in implementation efforts. Respondents make general statements about the construct manifesting in a positive way but without concrete examples: (1) the construct is mentioned only in passing or at a high level without examples or evidence of actual, concrete descriptions of how that construct manifests; (2) there is a mixed effect of different aspects of the construct but with a general overall positive effect; and/or (3) there is sufficient information to make an indirect inference about the generally positive influence.
+2	The construct is a positive influence in the organization, a facilitating influence in work processes, and/or a facilitating influence in implementation efforts. The majority of respondents describe explicit examples of how the key or all aspects of a construct manifests themselves in a positive way.
Missing	Respondent(s) were not asked about the presence or influence of the construct or, if they were asked about a construct, their responses did not correspond to the intended construct and were instead coded to another construct. Respondent(s)’ lack of knowledge about a construct does not necessarily indicate missing data and may instead indicate the absence of the construct.

To enhance trustworthiness [51], CV and ME analyzed data separately. Throughout the analysis, findings were examined and discussed among all authors until a common understanding of the final classifications of the coding categories and ratings into the constructs of CFIR was reached. To organize and manage the large amount of data [52], we used the software program NVivo version 9 (QSR International, Doncaster, Victoria, Australia). To increase the transparency of the interpretation, coding categories are illustrated with quotations in the presentation of the results subsequently.

## Results

### Evaluation of the Implementation of Internet-Based Patient-Provider Communication Using the Consolidated Framework for Implementation Research

The main results presented subsequently address identified barriers and facilitators influencing the implementation of IPPC in 5 hospital units using CFIR as the conceptual framework (aim 1) and the applicability of CFIR to identify determinants distinguishing between high and low implementation success (aim 2). As shown in Table 2, 6 constructs strongly distinguished between high and low implementation units. Six constructs weakly distinguished units and 16 constructs were mixed across units (Table 2). A description of findings on CFIR constructs follows subsequently.

**Table 2:** Ratings assigned to CFIR constructs by unit based on the rating criteria.^a^

Domains and constructs of CFIR		High implementation units				Low implementation unit	Distinguishing constructs^b^
		Unit 1	Unit 2	Unit 3	Unit 4	Unit 5	
**1. Intervention characteristics**							
	Intervention source	External	External	Missing	Missing	External	
	Evidence strength & quality	Missing	Missing	Missing	Missing	Missing	
	Relative advantage	+2	+2	0 (mix)	+1	0	Weak
	Adaptability	+1	+2	+1	+1	+1	
	Trialability	+2	+2	+2	+2	–1	Strong
	Complexity (reverse rated)^c^	+1	+1	+1	+1	+1	
	Design quality and packaging	+1	+1	+1	–1	0	
	Cost	0 (mix)	0	–1	–2	Missing	
**2. Outer setting**							
	Patient needs & resources	0	+1	0	0	–1	Weak
	Cosmopolitanism	Missing	Missing	Missing	Missing	Missing	
	Peer pressure	Missing	Missing	Missing	Missing	Missing	
	External policies & incentives	Missing	Missing	Missing	Missing	Missing	
**3. Inner setting**							
	Structural characteristics	0	0	0	0	–1	Weak
	Networks & communications	+1	0 (mix)	+1	+2	+1	
	Culture	–1	–1	+1	–1	–2	Weak
	Implementation climate						
	Tension for change	+1	+1	0 (mix)	+1	–2	Strong
	Compatibility	Missing	+1	0 (mix)	+2	–2	Strong
	Relative priority	0	Missing	0	+1	–2	Strong
	Organizational incentives & rewards	0	+1	0	Missing	Missing	
	Goals & feedback	Missing	Missing	Missing	0 (mix)	0	
	Learning climate	+1	+1	Missing	Missing	+1	
	Readiness for implementation						
	Leadership engagement	+1	0	+1	0	+1	
	Available recourses	+2	+2	+2	+2	–1	Strong
	Access to information and knowledge	+2	+1	+2	+2	Missing	
**4. Characteristics of individuals**							
	Knowledge and belief about the intervention	+2	+2	0 (mix)	+2	–1	Weak
	Self-efficacy	+1	+1	+2	+2	+1	
	Individual stage of change	–1	+2	–1	–1	–1	
	Individual identification with organization	Missing	Missing	Missing	Missing	Missing	
	Other personal attributes	Missing	Missing	Missing	Missing	Missing	
**5. Process**							
	Planning	Missing	+1	Missing	+1	–2	Strong
	Engaging						
	Opinion leaders	Missing	Missing	Missing	Missing	–2	
	Formally appointed internal implementation leaders	0	0	+1	0	–1	Weak
	Champions	Missing	Missing	Missing	Missing	Missing	
	External change agents	–1	+2	+1	+2	–1	
	Executing	Missing	Missing	Missing	Missing	Missing	
	Reflecting & evaluating	0	Missing	Missing	0	Missing	

^a^–2: Construct found to have a strong negative influence; –1: construct found to have a weak negative influence; 0: construct found to have neutral influence; 0 (mix): construct had mixed positive and negative influences, which balanced each other; +1: construct found to have a weak positive influence; +2: construct found to have a strong positive influence; Missing: not asked or miscoded.

^b^Weak: construct weakly distinguished between high and low implementation units; Strong: construct strongly distinguished between high and low implementation units.

^c^Reverse rated: a positive rating means a less complex implementation.

### Intervention Characteristics

#### Intervention Source (Not a Distinguishing Construct)

Regarding the respondents’ perception about whether the IPPC service was externally or internally developed, respondents in 3 units described the IPPC service as externally developed with the research center taking the initiative for the IPPC implementation. This was viewed as a barrier for the implementation of IPPC at the low implementation unit. They said that the engagement for IPPC could have been increased if the implementation had been internally initiated either from the hospital administration as a mandatory intervention or from a person who was known in the unit beforehand:

When someone comes from the outside, you know, it’s got nothing to do with the person, and there could well be a project, but it may well be that people would be more dedicated if it were someone they knew in the department who was running it, that they said: “Oh yes, the woman over there, she has a project, yes, we will help her.”

#### Relative Advantage (Weakly Distinguishing Construct)

Related to their professional responsibility, the respondents described the relative advantage of communication with the patients via IPPC compared with face-to-face and telephone communication within 4 areas: (1) quality improvement, in that they could provide a better service to their patients; (2) patient safety, by bridging information gaps and discovering serious symptoms; (3) knowledge development, in that IPPC could provide insight into patients’ problems and act as a repository for frequently asked questions and responses; and (4) time-saving and focused, in that they could answer patients’ messages without interruptions when they had available time. They said that IPPC was a supplement and could not be the only means of communication. A disadvantage described by staff at the low implementation unit was the lack of important nonverbal communication in IPPC: “We lose voice, tone of voice, and pitch as well as catching undertones, reading between the lines.” They said that IPPC was not suited for communication about complex issues, such as expected life span or complicated medication.

#### Adaptability (Not a Distinguishing Construct)

Respondents reported that the IPPC service had been well adapted to the staff’s other duties in all units. The respondents described how they answered messages from the patients and cooperated with the other IPPC team members. They wanted the patients to get an answer as soon as possible and they could become stressed if they could not give the patient the answer fast enough.

#### Trialability (Strongly Distinguishing Construct)

According to respondents in all units, the IPPC service was tested in a well-run and exciting study:

I knew that it was limited, but I had actually hoped that it could continue because the trend we saw was so positive, really, that it has worked very well.

However, at the low implementation unit there was a slightly negative perception of the way the study was performed and how the respondents perceived the study’s research questions:

I don’t know, if there were too many questions, there were too many quality indicators versus the quantitative data that we are used to.

#### Complexity (Not a Distinguishing Construct)

The IPPC service was described by all units as not very complex to implement. Factors that decreased implementation complexity included the limited number of patients and health care providers involved at each unit and the limited number of questions that the patients posted through IPPC. However, the shift from oral to written communication was described as challenging by respondents in the low implementation unit and by 2 of the high implementation units.

#### Design Quality and Packaging (Not a Distinguishing Construct)

At all units, IPPC was described as easy for the health care providers to use. However, there had been some log-in problems for both health care providers and patients related to server issues and a cumbersome log-in procedure.

#### Cost (Not a Distinguishing Construct)

The respondents at 4 units were focused on the units’ financial status and these respondents were conscious that funding was an issue for implementation of new interventions, such as an IPPC system, into clinical practice. Three high implementation units considered cost-effectiveness in terms of how many patients they thought should use IPPC before it was appropriate to offer it as a regular intervention and they said patient use in the current implementation was too low to introduce it as a regular service. However, some respondents said that if patients wanted it, and if those patients who used it were satisfied, they believed IPPC should nevertheless be offered as a regular service.

### Outer Setting

#### Patient Need and Resources (Weakly Distinguishing Construct)

Respondents from all high implementation units said that the IPPC service would benefit the patients. Therefore, they were surprised, and to some degree disappointed, that only a few patients used the IPPC service:

I’m a little disappointed that the patients didn’t use it [IPPC]. I think it’s sad that the patients didn’t appreciate the project more.

Nevertheless, they thought that patients felt the IPPC service was good to have even if they did not use it. Four explanatory factors for patients’ nonuse were described: (1) patient characteristics, such as diagnosis, health condition (too sick or too healthy), age, and eHealth experience; (2) not in need of IPPC due to not having any questions, experiencing a good follow-up in the regular service, or preferring other forms of communication; and (3) barriers, such as information overload, inappropriate treatment phase, cumbersome log-in, forgetting about the IPPC, too many forms to fill out as part of the study, or not wanting to disturb the health care providers. In the low implementation unit, respondents said that the patients at their unit had less need of the IPPC service than others because the patients received very close face-to-face and telephone follow-up, and that the frontline health care providers at the unit regarded it as too burdensome for the patients to participate in the IPPC study:

And they are patients with severe crisis reactions. It’s not important for them, you know, and then it gets difficult for us to come with this [IPPC] on top of everything else.

### Inner Setting

#### Structural Characteristics (Weakly Distinguishing Construct)

All units were specialized in their medical discipline and were staffed with highly qualified health care providers. The structure and organization varied between the units with respect to professional autonomy. At the low implementation unit and 2 high implementation units, the nurse management was peripheral and the frontline health care providers independently collaborated with one another at the unit. At the other 2 high implementation units, the management had a stronger presence in a hierarchical structure in the unit. For the low implementation unit, the peripheral nurse management had a negative impact on the implementation of IPPC (see Leadership Engagement).

#### Networks and Communication (Not a Distinguishing Construct)

Arenas for discussion of individual patients’ treatment and follow-up were described by all units. One of the high implementation units had a well-functioning team where all professional groups met every week and the IPPC system had been on the meetings’ agendas. In one of the other high implementation units, staff members felt they were working on their own with too few arenas for meetings between the different professional groups. In terms of operating the IPPC system, the procedures were viewed as suitable at all units with nurses coordinating the messages from the patients and the other professional groups answering messages when they received them from the nurses.

#### Culture (Weakly Distinguishing Construct)

A conservative culture toward implementation of new interventions was described by all units. The respondents said their units were slow to introduce new things and that health care providers liked to do things the way they always had done them:

It’s relatively conservative; we’re a bit protective about our own things.

Respondents at the low implementation unit also described many individualists and strong personalities among the health care providers, who did not want to be told to do something different. At this unit, they were used to conducting research studies; however, they did not have experience in research led by nurses and had a view of nursing research as not being “real.” This IPPC study had low status compared with clinical trials performed by physicians at the unit, which hindered some of the unit staff from taking the study seriously:

This wasn’t a medical study, after all. This was a nursing study and that’s something we have never worked with and that, I mean, there were no heavyweight pharmaceutical companies behind it and there were no heavyweight professors behind it [which was not true].

At one of the high implementation units, they described the culture as a little more open to new ideas explained by a young staff at the unit open to changes.

#### Implementation Climate

Six subconstructs in CFIR illuminate the implementation climate for IPPC and the units’ capacity for change:

1. Tension for change (strongly distinguishing construct), the perception of a need for a change, was reflected in the respondents’ view of IPPC as a possible future medium in health care. This was described by respondents in 3 of the high implementation units, which were enthusiastic about using IPPC: “Today, our everyday work is just getting even more challenging with all these phones ringing, so I’m convinced that this [IPPC] will need to be offered to everyone because it will ease people’s burden at work.” Respondents at the low implementation unit, however, did not express a need for IPPC, either for themselves or for patients. The respondents said they had well-established follow-up with patients face-to-face and by telephone: “I believe there is a great need for extra communication, much less here because we make such clear arrangements and they have an open telephone line to the nurses...So I guess that’s my conclusion, that perhaps our unit wasn’t the one with the greatest need.”
2. Compatibility (strongly distinguishing construct) between the health care providers involved and IPPC was expressed by 3 of the high implementation units in terms of being well adapted to the hospital’s overarching philosophy of being open and accessible to patients: “The basic concept [of IPPC] is very good, so I feel it fits very well with the profile of the hospital.” In the low implementation unit, respondents said that IPPC had poor fit because the patients had complicated problems, which meant that the health care providers needed to talk to the patients face-to-face anyway: “These medical questions; often they take a bit more follow-up than what one might be able to do by email, you might need to talk to the patient, and listen to the patient to find out how poorly are you really doing?” They said they had tried to communicate to the management that IPPC would not fit the unit and that the number of eligible patients originally estimated for the study was too high.
3. Relative priority (strongly distinguishing construct) for introducing patients to IPPC was described as good at 3 of the high implementation units. They talked about enrolling patients to IPPC in a neutral or positive way: “It was not so difficult to recruit patients; it actually went reasonably well.” At the low implementation unit, introducing IPPC to patients was poor. The general view was that staff did not want to put effort into the implementation of IPPC and, thus, omitted to inform most of the patients about IPPC: “It did not fit in, you know, and then suddenly the patient has left, and then you have forgotten about it, because there were so many other things, and then, demands from all sides, you know, and then this comes on top of everything else.”
4. Organizational incentives and rewards (not a distinguishing construct) were not given to either health care providers or units in the form of monetary rewards or other incentives. However, at one of the high implementation units, respondents felt proud to have the IPPC service and felt it could increase their professional status.
5. Goals and feedback (not a distinguishing construct) that the respondents had received during the IPPC service implementation were not much discussed at either of the units. Those who mentioned it did so in a neutral way, except one of the high implementation units, which expressed a wish for more feedback regarding the results from the questionnaires the patients had filled out as part of the study over the implementation period.
6. The learning climate (not a distinguishing construct) was described as good by both high and low implementation units. They aimed to offer evidence-based health care and the frontline nurses were offered nursing supervision and coaching.

#### Readiness for Implementation

The units’ commitment to the implementation of IPPC was illuminated in CFIR through 3 subconstructs:

1. Leadership engagement (not a distinguishing construct) was described as strong and involved in the early phases of the implementation of IPPC at both high and low implementation units, but respondents said that the managers had not followed up later during the implementation. Two of the respondents at the high implementation units said that their manager did not even know about the IPPC service, but that the respondents had an independent role and therefore did not miss it either: “So, to be perfectly honest, I haven’t had any contact with management regarding this project at all. I don’t know whether they are aware that I have been involved in the project.” At the low implementation unit, the nurse manager was peripheral, which the respondents described as negative because the unit did not have a manager who led the implementation. Even if the management had been enthusiastic about IPPC, the anchoring to the frontline health care providers was missing: “So the thing is this, if you are going to have a project, the whole department must agree on it, and that didn’t happen here. Because management thought this was great, but further down, they thought this was an extra burden.” The respondents expressed a lack of consistency between the nurse manager’s goal and the frontline health care providers’ perception of what was realistic to conduct.
2. Available resources (strongly distinguishing construct) were perceived as sufficient in terms of available time at all high implementation units: “We haven’t spent a lot of time on it...We have just answered in between the other things we do. We haven’t needed to have any time set aside to sit with it.” At the low implementation unit, respondents were worried about not having enough time for both patient recruitment and answering patient messages: “It does mean that you must have allocated time for it because you can’t just do it on the side, at least not all the time.”
3. Access to information and knowledge (not a distinguishing construct) in terms of information and training were expressed as satisfactory by all high implementation units.

### Characteristics of Individuals

The personal perception of IPPC of the respondents is illuminated through 3 subconstructs:

1. Knowledge and beliefs about the intervention (weakly distinguishing construct) were described with positive statements at the high implementation units where they were positive and enthusiastic about IPPC: “I feel very positive about this. I think it is a very good service.” They said that IPPC was modern and future-oriented and that they thought it should be a permanent service. One of the respondents at a high implementation unit had a different view than the others and preferred personal contact with the patients, so this unit was coded as a “mix” (0). At the low implementation unit, they said that the idea was good, but that IPPC would fit better into other parts of the health care system than their own unit. The respondents preferred personal contact with the patients: “I like the personal contact with the patient...And I sort of like having some eye contact and like to, there’s so much that also gets said in the pauses you know.”
2. Self-efficacy (not a distinguishing construct) was high at all units in that they were able to answer the questions from the patients without much difficulty.
3. Individual stage of change (not a distinguishing construct) was affected by the fact that the operation of IPPC was not yet fully incorporated. All felt some inconvenience using the IPPC because they seldom received messages. Only one of the respondents at a high implementation unit viewed IPPC as an integrated part of the daily work: “For me, it has in a way become like something ordinary in my everyday life. I have never thought that ‘oh goodness, this thing is the research project’.”

### Process

#### Planning (Strongly Distinguishing Construct)

At 2 high implementation units, they were satisfied with the planning of the study and by participating in meetings with the research center they felt that they were able to have an impact on the implementation: “It was very good for those who followed the meetings, that you felt you could influence something.” At the low implementation unit, not being part of the planning led to less engagement among the nurses involved and a more critical attitude to the implementation: “Perhaps all the nurses should have been more involved from the start, so that everyone was prepared to be included and to share the load, and then I think one would have been more committed to finding these patients and more dedicated to the study, because I felt that was lacking.”

#### Engaging

The engagement of different actors in the implementation of IPPC affected the process in different ways:

1. Opinion leaders (not a distinguishing construct) were illuminated only in the low implementation unit and there they had had strong negative views of IPPC, which affected the entire unit: “And then there may be some strong personalities who send signals, that is, who are highly verbal, and then that spreads around a bit in the unit...The counterculture isn’t so strong when someone is obviously waving the negative flag.”
2. Formally appointed implementation leaders (weakly distinguishing construct) were specially selected by their managers to operate the IPPC system based on their experience, role, and position in the unit: “There were a handful who were picked out for it, based a little bit on having worked for a few years and getting some experience and knowing the patient group and so on. I don’t think I would assign a newcomer to this, no.” At all units except the low implementation unit, the selected nurses felt comfortable that they had been chosen to operate the IPPC system. At the low implementation unit, respondents described that the other frontline health care providers in the unit were highly independent and that the formally appointed implementation leaders were put in a difficult position because they did not have the authority to instruct the other nurses and physicians what to do: “It was up to the individual nurse to take responsibility for and ask [the patient about IPPC]. For us, that’s the way it is with everything. And then I didn’t have capacity to go and ask ‘have you asked?’ I mean, that’s not how it works...No, I don’t want to take on that kind of role, no. That would be wrong.”
3. External change agents (not a distinguishing construct) in this implementation were the research center that was responsible for the implementation and managing the study. Three high implementation units were satisfied with the follow-up from the research center, except one of the respondents who did not feel informed about being a part of the research study and participating in interviews, but thought the only task was to answer some messages in the IPPC. The last high implementation unit and the low implementation unit said that the person from the research center had changed in the middle of the process and that this influenced the process negatively because the continuity was lost. At the low implementation unit, respondents also said that the person from the research center should have been in the unit more frequently to push the process.

#### Reflecting and Evaluating (Not a Distinguishing Construct)

At 2 high implementation units, respondents looked forward to receiving a summary from the research center about how the patients viewed having a IPPC system and also how the other units had operated the IPPC system and their view on it.

### Comparison of Distinguishing Factors Across Studies

To identify distinguishing factors across studies (aim 3), we compared the 12 CFIR constructs found in our study that distinguished between high and low implementation units with the results from 2 other studies that also found constructs distinguishing between high and low implementation units (Table 3). One of the studies aimed to explain the variation in implementation success of MOVE!, a program for obesity management in medical centers and community-based outpatient clinics. This is also the study of the CFIR developers on which we based our analysis method [50]. The other study aimed to describe variation in implementation of California’s Full Service Partnerships, a service delivery model for supported housing among persons with serious mental illness [43]. Across the 3 studies, 24 of 39 CFIR constructs distinguished between high and low implementation units in at least one of the studies. Eleven constructs distinguished between units in 2 of 3 studies. Two constructs distinguished between units in all 3 studies. Constructs that did not distinguish between high and low implementation units were reported in only one of the other studies with which we compared our results [50]. Across that study and our study, 8 constructs did not distinguish between high and low implementation units in both studies and 15 constructs did not distinguish between high and low implementation units in one of the studies (Table 4).

**Table 3:** CFIR constructs distinguishing between high and low implementation units across studies that reported them.

Domains and constructs of CFIR		Damschroder & Lowery [50]	Gilmer et al [43]	Our study	Studies with overlapping distinguishing constructs, n
**1. Intervention characteristics**					
	Relative advantage	Yes		Yes	2
	Trialability			Yes	1
**2. Outer setting**					
	Patient needs & resources	Yes	Yes	Yes	3
	Cosmopolitanism		Yes		1
	External policies & incentives	Yes	Yes		2
**3. Inner setting**					
	Structural characteristics			Yes	1
	Networks & communications	Yes	Yes		2
	Culture		Yes	Yes	2
	Implementation climate				—
	Tension for change	Yes		Yes	2
	Compatibility		Yes	Yes	2
	Relative priority	Yes		Yes	2
	Goals & feedback	Yes			1
	Learning climate	Yes			1
	Readiness for implementation				—
	Leadership engagement	Yes	Yes		2
	Available recourses	Yes	Yes	Yes	3
	Access to information and knowledge		Yes		1
**4. Characteristics of individuals**					
	Knowledge and beliefs about the intervention		Yes	Yes	2
	Other personal attributes		Yes		1
**5. Process**					
	Planning	Yes		Yes	2
	Engaging				—
	Opinion leaders		Yes		1
	Formally appointed internal implementation leaders		Yes	Yes	2
	Champions		Yes		1
	External change agents		Yes		1
	Reflecting & evaluating	Yes			1
Distinguishing constructs in each study, n		12	15	12	

**Table 4:** Constructs nondistinguishing between high and low implementation units across studies that reported them.

Domains and constructs of CFIR		Damschroder & Lowery [50]	Our study	Studies with overlapping nondistinguishing constructs, n
**1. Intervention characteristics**				
	Intervention source	Yes	Yes	2
	Evidence strength & quality	Yes		1
	Adaptability	Yes	Yes	2
	Trialability	Yes		1
	Complexity	Yes	Yes	2
	Design quality and packaging	Yes	Yes	2
	Cost	Yes	Yes	2
**2. Outer setting**				
	Cosmopolitanism	Yes		1
	Peer pressure	Yes		1
**3. Inner setting**				
	Networks and communication		Yes	1
	Implementation climate			—
	Compatibility	Yes		1
	Organizational incentives and rewards	Yes	Yes	2
	Goals and feedback		Yes	1
	Learning climate		Yes	1
	Readiness for implementation			—
	Leadership engagement		Yes	1
	Access to information and knowledge	Yes	Yes	2
**4. Characteristics of individuals**				
	Self-efficacy		Yes	1
	Individual stage of change		Yes	1
**5. Process**				
	Engaging			—
	Opinion leaders		Yes	1
	Formally appointed internal implementation leaders	Yes		1
	Champions	Yes		1
	External change agents	Yes	Yes	2
	Reflecting and evaluating		Yes	1
Nondistinguishing constructs in each study, n		15	16	

## Discussion

### Evaluation of the Implementation of Internet-Based Patient-Provider Communication in Five Hospital Units

We identified barriers and facilitators influencing the implementation of IPPC in 5 units at a university hospital by means of the CFIR framework (aim 1). We found 12 constructs distinguishing between high and low implementation units. Half of the distinguishing constructs were related to the inner setting domain of CFIR and showed that structural characteristics of the units, available resources, culture, and implementation climate influenced the implementation of IPPC. Thus, our results support claims that the context requires attention in itself and not only as a background description of a study [24].

This study also found that the constructs relative advantage, patient needs and resources, and knowledge and belief about the intervention were tied together, and that they distinguished between high and low implementation units. This indicates that the health care providers’ perception of IPPC as useful either for themselves in their professional work or for the patients affects the implementation. This is consistent with another study that found that clinical need and usefulness of an intervention may be crucial factors for successful implementation [53]. In information technology implementation, addressing the technology is also recommended [33] but, in this study, IPPC was regarded neither as complex nor technically challenging. This might imply that the usability of the IPPC system was good or that the professionals involved had high technical skills or both and, thus, technology demanded less attention.

Finally, the process domain included constructs distinguishing between high and low implementation units in this study and revealed that, in particular, the planning phase of an implementation is critical. In addition, the engagement of health care providers who are enthusiastic and can support the implementation throughout the process proved important for the implementation outcome. In this implementation, it was up to the individual nurses whether the patients were offered IPPC or not. Automatically offering all patients information about IPPC in a standardized introduction might have increased the number of patients enrolled in the study. By identifying and addressing the barriers beforehand, the implementation interventions could be tailored to meet and overcome the barriers toward a more successful implementation outcome [54]. If nurses at the low implementation unit had been more involved in the planning phase, it would likely contribute to more insights about the unit and the health care providers working there. Interventions that are tailored to prospectively identified barriers are more likely to improve practice [55].

### Consolidated Framework for Implementation Research’s Ability to Distinguish Between High and Low Implementation Success

The CFIR provides a comprehensive overview of all aspects that can affect implementation and was helpful in creating the interview guide to ensure that all relevant aspects were covered in the interviews. However, the strength of CFIR as broad and comprehensive can also be its weakness. In our study, we had a number of constructs that were not illuminated through the interviews because the allotted time for the interviews did not allow for all constructs to be illuminated. Others have also found that the number of CFIR domains covered in a single study varies between 4 and 25 [36,56]. Even in the study of Damschroder and Lowery [50], where this method is described, the CFIR developers reported constructs that were not addressed. Therefore, CFIR may be too broad to capture all constructs through one set of individual interviews. By trying to capture the “big picture” in as many domains as possible, the depth and specificity in terms of details that may be crucial for a successful implementation may be lost. To capture all aspects of CFIR, a series of interviews may be needed. In contrast, a focus on specific facilitators or barriers, with a limited sample of sites or participants, would benefit from choosing a restricted sample of constructs that had been shown to distinguish between successful and weak implementation outcomes. Small studies can target discrete but significant questions and thereby speed knowledge translation [57].

The CFIR is institution-centric in that patients are placed under the outer setting domain, thus indicating their peripheral role in the implementation process. Although this is natural for many traditional implementations, it is somewhat unintuitive for patient-centric interventions. With the increasing emergence of patient-centric models of care, including models for homes and communities [58-60], it may be worth exploring implications for the appropriateness of all current CFIR domains. For example, patients are given only one single construct, intended to capture both their needs and their resources. In comparison, the health care providers have 2 whole domains, both the inner setting domain with 12 constructs and characteristics of individuals with 5 constructs. This applies not only for CFIR; others also have pointed out the need for greater consideration of the patient role in implementation theory [32,33,42].

Some of the constructs of CFIR are concrete and relatively easy to measure, whereas others are broad and abstract and more difficult to capture. Some of the constructs in the domain of characteristics of individuals were missing in our study (individual identification with organization and other personal attributes) and the same is seen in other studies using CFIR [39,61]. The question then is if these constructs are providing new information or if they are overlapping with other constructs. Probably they would have been used more if CFIR developers [31,50,62] had described and explained them more specifically.

### Consolidated Framework for Implementation Research Use Across Studies

To increase the knowledge about CFIR’s applicability to compare implementation across settings and interventions, we compared our results with results from 2 other studies that also found CFIR constructs that distinguished between high and low implementation units (Table 3) or did not distinguish between them (Table 4).

In all 3 studies, the CFIR constructs patient need and resources and available resources distinguished between high and low implementation units. This indicates that factors essential for successful implementation is the health care providers’ belief in the intervention as useful for their patients and their experience of having the necessary resources to make use of the new intervention. Eight of 12 distinguishing constructs in our study also appeared in one of the 2 other studies. This may suggest that in total 10 of the constructs we found in our study deserve particular attention because they also appeared in settings and for interventions quite different from ours.

Three distinguishing constructs were found in the 2 other studies that did not appear in our study. One of them, leadership engagement, is pointed out as important for successful implementation in several other studies [63,64]. One possible reason for the absence in our study is that many of the units in our study were staffed by nurses who were working independently led by their own self-management rather than by a unit manager. Therefore, there is no reason to question the importance of leadership even if it did not occur in our study. Inner motivation and own belief and perceived advantage of the intervention for the professionals involved and their patients played a more crucial role than leadership engagement. This may indicate that the characteristics of individuals are more influential when the management is peripheral. This is consistent with another study that found that management was not always necessary if the planning and conducting of the implementation were taken care of by other means [17].

Constructs that did not distinguish between high and low implementation units were obtained from only 2 studies because it was not addressed in the third study and, therefore, they are too limited to draw conclusions about. However, across the 2 studies, most of the nondistinguishing factors were related to the intervention characteristics domain, indicating that implementation success was related to other aspects than the intervention itself.

### Strengths and Limitations

There are a number of limitations to our study. The study was conducted at a single university hospital and the results may not be representative of other practice settings. However, the inclusion of 5 units and comparisons across the units, which revealed that there were clear differences among the units, increases the transferability to other settings.

There is a limitation to the study in that it compares 4 high implementation units to only one low implementation unit. With only one low implementation unit, it is difficult to know whether its characteristics can be representative of other low implementation contexts or whether they are merely idiosyncratic to that one unit. However, the majority of CFIR constructs distinguishing between high and low implementation units in our study were also shown in other studies (Table 3) indicating that the characteristics for low implementation units are present across studies.

Another weakness of this study is that we were not able to present the exact number of available patients from all 5 units who could have been offered information about IPPC. At 2 units there were too many health care providers involved in the identification and first information about IPPC to the patients, so we did not manage to develop a complete reporting routine for patients who were not approached. However, the difference between the high and low implementation units regarding patients offered information about IPPC was prominent and also supported through the interviews.

Another limitation in this study is that one respondent did not allow use of a voice recorder during the interview and that another preferred to respond in writing. Even if these 2 respondents did not want to be included in the interview procedure designed for the study, their contributions were considered too crucial for the study to be omitted based on the departure in methodology. However, a strength of the study is that it includes all the health care providers who had played an active role in the implementation of IPPC and all who were asked were also willing to participate.

### Suggestions for Further Research

Next step recommendations for CFIR research are to continue comparing high and low implementation units. In addition, longitudinal studies could provide insight into how an implementation process evolves over time and which factors are of special importance during the different phases of an implementation. Further, in light of increases in patient-centric models of care, we suggest to strengthen the patient-related constructs. These types of studies could help develop a next edition of the CFIR framework with refined parsimony and testability of its constructs. Finally, adding practical guidelines for implementation based on CFIR will make the framework more user-friendly not only for researchers, but also for the health care providers who are conducting implementations in clinical practice.

### Conclusion

This study adds insights into the barriers and facilitators influencing the implementation of an IPPC system and the differences between high and low implementation units by using the CFIR framework. We found 12 CFIR constructs distinguishing between high and low implementation units in our study, most from the inner setting domain, indicating that institutional factors are of particular importance for the implementation success in the given context. The health care providers’ belief in the intervention as useful for themselves and the patients and the conduct of the implementation process, including engagement of key personnel, were also identified as important for the implementation of IPPC.

Comparison of CFIR constructs across 3 studies identified 2 constructs as particularly important in all 3 studies (patient needs and resources and available resources of health care providers) and an additional 11 constructs in 2 studies. CFIR was helpful in guiding the study and ensuring that all main aspects were covered during the interviews. Although CFIR’s strength is being broad and comprehensive, this also limits its usefulness because it does not distinguish between the relative importance of its many constructs. This is the first study to identify which constructs in the CFIR are the most promising to distinguish between high and low implementation success. Thus, this study can contribute to the refinement of CFIR to become a more succinct and parsimonious framework for planning and evaluation of eHealth implementation studies.

## Abbreviations

CFIR: Consolidated Framework for Implementation Research
IPPC: Internet-based patient-provider communication
PARiHS: Promoting Action on Research Implementation in Health Services
RE-AIM: Reach Effectiveness Adoption Implementation Maintenance
